# Fecal microbiota transplantation: can it circumvent resistance to PD-1 blockade in melanoma?

**DOI:** 10.1038/s41392-021-00585-5

**Published:** 2021-05-08

**Authors:** Lisa Derosa, Laurence Zitvogel

**Affiliations:** 1 Gustave Roussy Comprehensive Cancer Institute, Clinicobiome, Villejuif, France; 2 Faculty of Medicine, Université Paris Saclay, Le Kremlin-Bicêtre, France; 3 INSERM U1015, Villejuif, France; 4 Equipe labellisée par la Ligue contre le cancer, Villejuif, France; 5 Center of Clinical Investigations in Biotherapies of Cancer (CICBT) BIOTHERIS, Villejuif, France; 6 Suzhou Institute for Systems Medicine, Chinese Academy of Medical Sciences, Suzhou, China

**Keywords:** Cancer, Predictive markers

In two recent articles, Baruch et al.^1^ and Davar et al.^2^ bring up the first proof-of-concept that fecal microbiota transplantation (FMT) transfer clinical benefit in metastatic melanoma patients primarily resistant to immune checkpoint blockade (ICB). FMT shifted the microbiota of recipients toward a donor-type taxonomic composition associated with immune activation, anti-inflammatory tonus, and changes in the host metabolism.

The intestinal microbiota regulates many seminal functions of our meta-organism and accumulating evidence pointed to cause–effect relationships between a reduced diversity of the bowel commensalism or a deviated taxonomic composition of the microbiota and pathophysiological failures such as type 2 diabetes and inflammatory bowel disease (IBD). FMT is the most direct therapeutic intervention to eliminate a pathogen and/or transiently change the gut composition, as shown by the clinical effectiveness against recurrent *Clostridium difficile* infection^3^ and IBD^4^.

**Fig. 1:**
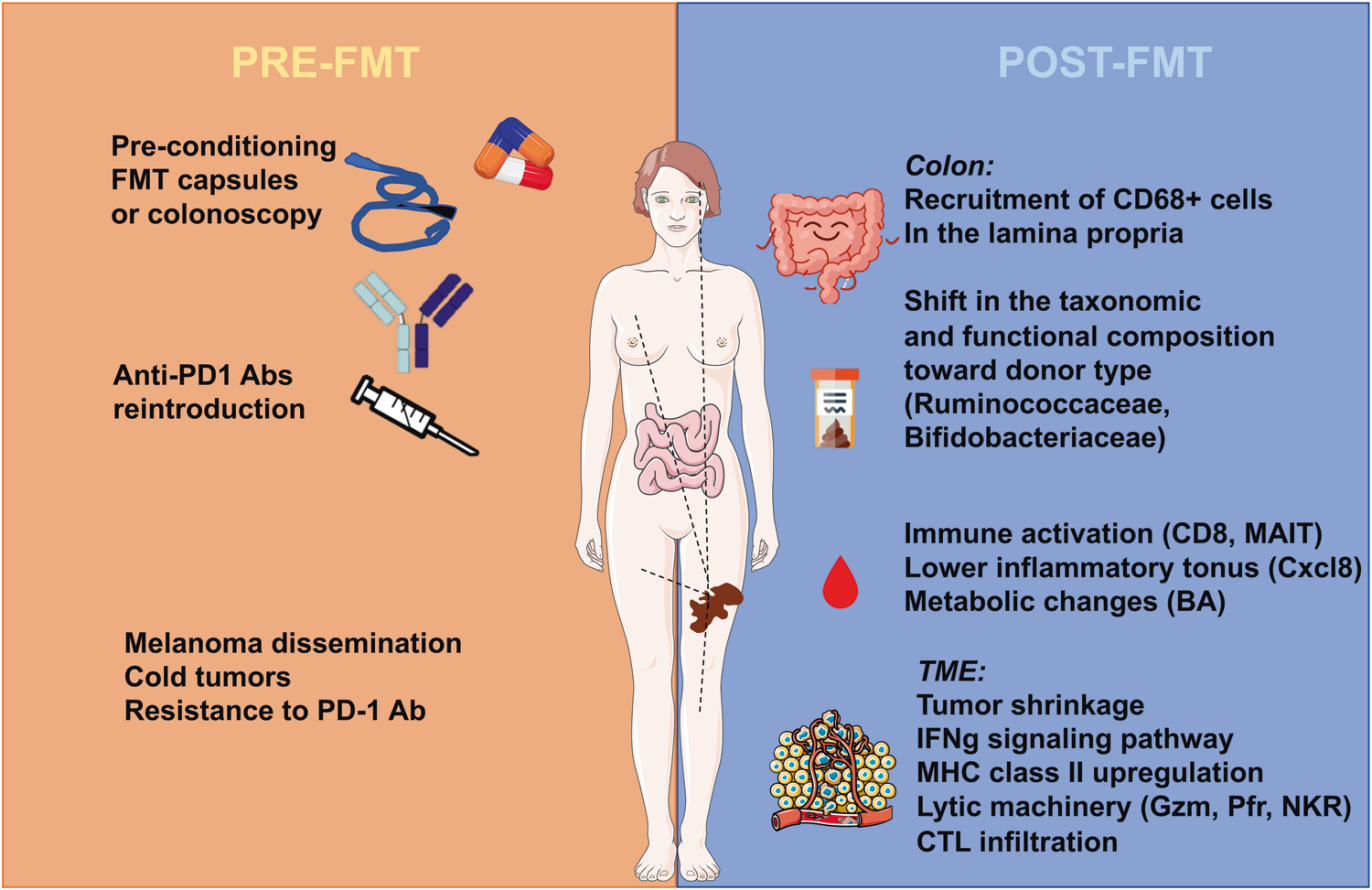
First proof-of-concept of the curative potential of FMT combined to reintroduction of anti-PD-1 Ab in cancer patients resistant to immunotherapy in two independent study. Nine different donors diagnosed with metastatic melanoma in partial or complete remission donated their stools for FMT (via oral or colonoscopic delivery) into 26 metastatic melanoma patients refractory to PD-1 blockade. The fecal product of distinct donors successfully engrafted in recipients who benefited the FMT and reprogrammed the local mucosae, reinstated tumor immunosurveillance, and induced changes in the metabolic, immune, and inflammatory tonus in nine patients who experienced long-term responses to this combinatorial regimen. Abbreviations: BA: biliary acids; CTL: cytotoxic T lymphocyte; FMT: fecal microbial transplantation; Gzm: granzymes; IFNγ: interferon gamma; MAIT: mucosal-associated invariant T cells; MHC: major histocompatibility complex; NKR: killer activating receptors; Pfr: perforin; TME: tumor microenvironment

There is a growing interest in manipulating the intestinal microbiota composition in the field of immuno-oncology since 2018^5^, when antibiotics (ATBs) were found associated with primary resistance to ICB. Moreover, FMT from non-responding (NR) cancer patients into tumor-bearing mice conferred the resistance phenotype to the recipient, whereas oral gavage of a responding (R) stool could restore responsiveness to PD-1 blockade^5^. These experiments supported the rationale to treat cancer patients who failed a first- or second-line immunotherapy with FMT derived from an R patient (Fig. 1).

Baruch et al.^1^ in Israel and Davar et al.^2^ in the United States conducted pioneering phase I trials, to evaluate the feasibility, safety, and efficacy of an FMT derived from R patients to reset the tumor microenvironment (TME) of 10 and 16 melanoma patients resistant (NR) to anti-PD-1 therapy respectively.

In the first study^1^, patients were treated with vancomycin and neomycin to deplete their own native microbiota before they were administered a lyophilized solution of fecal material from a donor into their gut via colonoscopy and via oral capsules. FMT was followed by the reintroduction of anti-PD-1 in combination with FMT every 14 days for a total of 90 days. Donations of fecal material originated from two R melanoma patients (donor 1 and donor 2) who achieved a durable (>1 year) complete response (CR). Although three of the five patients who received transplant from donor 1 exhibited objective responses including one CR, none of the five patients with transplants from donor 2 responded. In addition, the three responders crossed the progression-free survival landmark of 6 months. Only one recipient had a mild temporary bloating considered as an FMT-related adverse event. Donor 1 fecal specimen contained a high number of health-related bacteria (Lachnospiraceae and Eubacteriaceae family members, and bacteria of the Bifidobacteriales and Coriobacteriales order). Post treatment, all patients had changes in their gut microbiota and patients who received the more successful donor sample had greater relative abundance of *Ruminococcus* spp. (*R. gnavus* and *R. callidus*) and *Bifidobacterium adolescentis*, which are considered potentially favorable for immunotherapy, whereas those who received the donor 2 sample had overrepresentation of Clostridiaceae family members, associated with resistance to ICB in ATB-treated individuals. When comparing R and NR within donor 1 FMT recipients, the authors observed a higher relative abundance of *Enterococcus spp*. (previously described as immunogenic) and *Streptococcus australis*, and a lower representation of *Veillonella atypica* (reported as pro-TH17 and associated with dismal prognosis lung cancers) in R. These gut compositional shifts were associated with reprogramming of the TME with four patients from the same donor 1 exhibiting an upregulation of IFNγ-mediated signaling pathway and effector T functions.

In the second study^2^, 16 patients received a single FMT delivered by colonoscopy harvested from 7 different donors in complete or partial remission. A clinical benefit was conferred in six patients (three partial response (PR) and three stable disease (SD) > 12 months). Shot gun metagenomics profiling further revealed that the R recipient microbiota exhibited a significant shift toward the donor composition compared to the NR, which coincided with increased IgG responses against donor microbes. Successful FMT were enriched in *Ruminococcaceae*, *Bifidobacteriaceae*, and *Lachnospiraceae*, at the expense of *Tannerellaceae*, *Sutterellaceae*, and *Bacteroidaceae*. R displayed a higher frequency of blood CD8^+^CD56^+^ and activated mucosal-associated invariant T cells, both subsets overexpressing killer molecules coinciding with the upregulation of MHC class II molecules in tumor-infiltrating lymphocytes and decreased inflammatory markers in the plasma. Significant metabolic changes characterized R patients post FMT. Altogether, these studies confirmed that allogeneic FMT can shift the microbiome of a cancer recipient upon successful engraftment and reprogram the immune and inflammatory tonus of the host, resulting in a clinical benefit to ICB. Overall, the favorable safety profile and early signs of efficacy support further investigations on the use of FMT from R patients to restore the effects of immunotherapy in NR patients.

However, a number of questions are still unsolved. First, the mechanisms of action of FMT in immuno-oncology remain an open corundum. Chronic carcinogenesis and related medications may be associated with epithelial barrier dysfunction, creating or maintaining a gut dysbiosis. FMT would then be capable of restoring—at least transiently—a healthy microbiome defined by a broad diversity of commensals contributing to tissue repair, fitness of the intestinal epithelium, and maturation of the mucosal immune system. How the histological type of malignancy matters to affect the microbiota composition remains unclear. Hence, the extent to which this pioneering clinical success obtained in melanoma will be generalized to other tumor types has to be addressed in future studies. Alternatively, FMT could transfer distinct commensals endowed with immunostimulatory functions^5^, reinstating antitumor immune responses during concomitant immunotherapy. Likewise, the immuno-oncomicrobiome field needs novel diagnosis tools capable of accurately mapping the microbiota repertoire, to anticipate resistance to PD-1 blockade, to administer a compensatory microbiota-centered therapeutic intervention (MCI) and, reciprocally, FMT-suitable donors. Second, FMT may benefit from host conditioning (by narrow spectrum ATB or osmotic medications) or specific diet and prebiotics facilitating colonization of a diverse array of commensals. Third, one can surmise that successful anticancer therapies may also participate in restoring a healthier microbiota. We anticipate that the field of FMT will be soon rekindled by dysbiosis diagnosis kits, optimal delivery systems for prolonged bioactivity, appropriate pharmaco-kinetics, and dynamics tools, to improve outcomes with MCI in cancer patients.
